# Decision-making algorithm for TS in the ICU

**DOI:** 10.1186/cc14295

**Published:** 2015-03-16

**Authors:** L Marullo, A Tavano, P Fusco, F Ferraro

**Affiliations:** 1 Second University of Naples, Italy

## Introduction

Nowadays more percutaneous dilatational tracheostomy (PDT) methods are in use, but there is no ideal risk-free technique. We have outlined a decisional algorithm to choose the most appropriate technique in each case to reduce the incidence of complications.

## Methods

A retrospective review was performed using data from the last 14 years. Two hundred patients were selected. Patients were divided into two groups: one including the first 100 PDTs treated without the algorithm (nA-group) and the other including the last 100 patients treated with the algorithm (A-group). Valuation of clinical and anatomical features of the patients, neck ultrasound and fibrobronchoscopy came before the procedure [1]. The algorithm was formulated by our experience with PDT techniques, comparing the specific characteristics of each one with the physiopathological characteristics of each patient.

## Results

**Figure 1:**
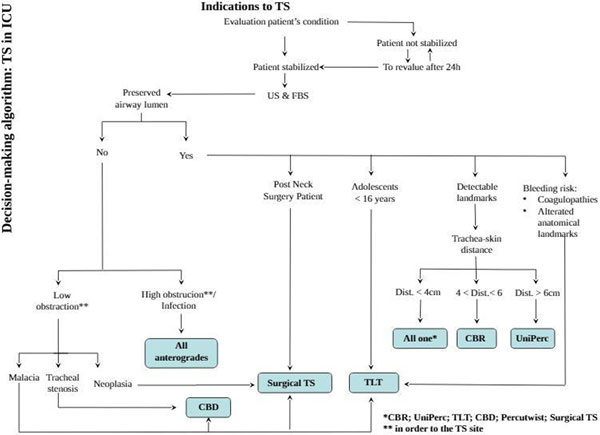


We recorded complications (bleeding, tracheoesophageal fistula, subglottic stenosis, tracheal rings' fracture, difficulty of placement, change of procedure) related to PDTs performed with and without applying the algorithm. We considered complications that occurred in our experience and we changed our modality in technique choice (Figure 1). Compared with the complications reported in the nA-group, use of the algorithm as a guide to choose the kind of PDT technique seems to reduce the incidence of complications (37% vs. 19%; *P *= 0.001 chi-square test).

## Conclusion

In our experience the application of the proposed algorithm may reduce the incidence of complications related to PDT in the ICU. However, a randomized controlled multicenter study would be necessary in order to confirm the efficiency and validity of the proposed algorithm.
